# Identification of QTLs for brown spot resistance in rice

**DOI:** 10.1270/jsbbs.25030

**Published:** 2025-08-20

**Authors:** Yuya Ota, Kengo Matsumoto, Yuto Honda, Satomi Ohashi, Daisuke Nakamura, Ritsuko Mizobuchi, Hiroyuki Sato

**Affiliations:** 1 Mie Prefecture Agricultural Research Institute, 530 Ureshinokawakita, Matsusaka, Mie 515-2316, Japan; 3 Institute of Crop Science, National Agriculture and Food Research Organization (NARO), 2-1-2 Kannondai, Tsukuba, Ibaraki 305-8602, Japan

**Keywords:** *Oryza sativa* L., brown spot, disease resistance, QTL

## Abstract

Rice brown spot (BS), caused by *Bipolaris oryzae*, is the third most prevalent rice disease in Japan and causes yield losses. In this study, quantitative trait loci (QTLs) associated with BS resistance were identified from the *indica* cultivars ‘Tupa 121-3’, ‘Naba’, and ‘IR58’. Two QTLs, *qBSR6-kt* (from ‘Tupa 121-3’) and *qBSR11-kn* (from ‘Naba’), were detected through QTL analysis of two F_2_ populations derived from crosses between the BS-susceptible *japonica* cultivar ‘Koshihikari’ and two resistant lines selected from chromosome segment substitution lines with chromosome segments from ‘Tupa 121-3’ and ‘Naba’ in the ‘Koshihikari’ genetic background. Another three QTLs from ‘IR58’ (*qBSR2-im*, *qBSR11-im*, and *qBSR12-im*) were detected by QTL analysis of recombinant inbred lines derived from a cross between ‘IR58’ and the BS-susceptible *japonica* cultivar ‘Mienoyume’. Among these detected QTLs, *qBSR6-kt*, *qBSR11-kn*, and *qBSR11-im* were confirmed to confer effective BS resistance in *japonica* rice cultivars. The two QTLs *qBSR11-kn* and *qBSR11-im* were located near the previously reported BS resistance QTL *bsr1* on chromosome 11. BS resistance QTL *qBSR6-kt* (on chromosome 6) was identified as a novel QTL that confers effective resistance to BS and may be valuable for pyramiding with *bsr1* or other BS resistance QTLs.

## Introduction

Rice brown spot (BS) is a fungal disease caused by *Bipolaris oryzae*, which infects various parts of rice plants (e.g., seedlings, leaves, and panicles). Barnwal *et al.* (2013) reviewed that the grain yield loss by BS ranged from 4 to 52% and the improvement of soil mineral deficiencies such as nitrogen, phosphorus, and potassium was effective against BS. The optimal temperature range for BS infection and lesion expansion is 27–30°C (Barnwal *et al.* 2013), and it is highly possible that BS will become a more serious disease under global warming (Mizobuchi *et al.* 2016).

In Japan, BS infected 146,204 ha, the third-largest area after rice blast (540,618 ha) and sheath blight (519,975 ha), in 2022 (Japan Plant Protection Association 2023). For farmers, the use of BS-resistant cultivars is more economical than spraying fungicides. Our research group has developed the cultivar ‘Mienoyume BSL’ (Matsumoto *et al.* 2021) and the line ‘Kanto IL 31’ (Mizobuchi *et al.* 2024), both of which possess BS resistance conferred by a quantitative trait locus (QTL) for BS resistance (*qBSfR11*, renamed *bsr1*). ‘Mienoyume BSL’ is a near-isogenic line (NIL) of the *japonica* cultivar ‘Mienoyume’, which is susceptible to BS. In Mie Prefecture, Japan, growers have been replacing ‘Mienoyume’ with ‘Mienoyume BSL’ since 2022. It has practical resistance to BS and higher yield than ‘Mienoyume’ in severe-BS fields, but lower yield in severe-BS fields than in mild-BS fields. Thus, the further enhancement of BS resistance by pyramiding multiple QTLs or genes is required.

In addition to *bsr1*, other QTLs for BS resistance have been reported (reviewed by Mizobuchi *et al.* 2016). Our research group has identified resistance QTLs derived from the *indica* cultivars ‘CH45’ (Matsumoto *et al.* 2017b) and ‘Dawn’ (Ota *et al.* 2021), which have high resistance to BS. As a result of evaluating resistance to BS within NIAS core collections of Japanese rice landraces and world rice, we found several BS-resistant cultivars (Matsumoto *et al.* 2017a). The objective here was to identify QTLs for BS resistance in these resistant cultivars. By using chromosome segment substitution lines (CSSLs) in which chromosome segments from two BS-resistant cultivars, ‘Tupa 121-3’ and ‘Naba’, were introduced into the ‘Koshihikari’ genetic background, we detected QTLs for BS resistance. In addition, we detected QTLs derived from *indica* cultivar ‘IR58’, which had the highest resistance among the materials in the NIAS core collections.

## Materials and Methods

### Plant materials for QTL analysis and confirmation of QTL effects

To select BS-resistant lines, we used two sets of CSSLs with chromosome segments from two BS-resistant *indica* cultivars, ‘Tupa 121-3’ (“KT-CSSLs”) and ‘Naba’ (“KN-CSSLs”), in the genetic background of the susceptible *japonica* cultivar ‘Koshihikari’ for field evaluation with four replications in 2018. These CSSLs were developed by Nagata *et al.* (2023), and genotype information for each CSSL is provided. To dissect BS resistance, we used two sets of 120 F_2_ plants derived from crosses ‘Koshihikari’/‘SL2524’ (one of the KT-CSSLs) and ‘Koshihikari’/‘SL3237’ (one of the KN-CSSLs). In 2020, the two F_2_ populations were evaluated for BS resistance in field trials with three replications for use in QTL analysis. We also used six sub-CSSLs (‘1-6’, ‘1-16’, ‘2979’, ‘2980’, ‘2-11’, and ‘3-2’) selected from sibling lines of ‘SL2524’ and eight sub-CSSLs (‘4-9’, ‘4-11’, ‘8-15’, ‘7-8’, ‘5-20’, ‘6-18’, ‘7-6’, and ‘3034’) selected from sibling lines of ‘SL3237’. These sub-CSSLs contained different simple sequence repeat (SSR) alleles near putative resistance QTLs. In 2020, these sub-CSSLs were grown with three replications to confirm the effects of detected QTLs for BS resistance.

We also used 90 recombinant inbred lines (RILs) in the F_5_ generation and 107 RILs in the F_6_ generation derived from the cross ‘IR58’ (resistant)/‘Mienoyume’ (susceptible). In the F_5_ generation, 17 of the 107 lines could not be evaluated for BS resistance owing to their poor germination. RILs were developed by the single-seed-descent method from different F_2_ plants. BS resistance of RILs in the F_5_ generation in 2018 and F_6_ in 2019 was evaluated in field trials with two replications for QTL analysis. The parent cultivars, ‘IR58’ and ‘Mienoyume’, were also evaluated with eight replications in both years. To confirm the effects of QTLs for BS resistance detected by QTL analysis of the RILs, we developed NILs in which ‘IR58’ alleles were introduced into the genetic background of ‘Mienoyume’. Marker-assisted selection (MAS) was conducted in the BC_3_F_2_ generation to select the NILs with the chromosomal fragments containing each QTL from ‘IR58’ by using SSR markers upstream and downstream of each of three putative QTL regions: RM13958 and RM3850 on chromosome (chr.) 2, RM5349 and RM27073 on chr. 11, and RM28305 and RM3331 on chr. 12 (McCouch *et al.* 2002). All NILs were confirmed to have ‘Mienoyume’ alleles at the QTL regions on chrs. 6 and 9 by using SSR markers RM20366 and RM6235, respectively. We developed three groups of NILs, designated MY2, MY11, and MY12, which had a single QTL derived from ‘IR58’ on chrs. 2, 11, or 12, respectively. Three NILs were selected from a BC_3_F_2_ individual in each NIL group. MY2 and MY11 NILs in the BC_3_F_3_ generation were crossed to develop three pyramided lines carrying QTLs on chrs. 2 and 11, designated as NIL group MY2+11. The recurrent cultivar ‘Mienoyume’ was also represented by three lines derived from three individuals. In 2024, BS resistance of NILs in the BC_3_F_4_ (MY2, MY11, and MY12) and F_3_ (MY2+11) generations was evaluated in field trials with three replications, along with their recurrent (Mienoyume) and donor (IR58) cultivars.

### Field trials

Field trials followed the method of Matsumoto *et al.* (2016). BS resistance was evaluated in the paddy field at Mie Prefecture Agricultural Research Institute (Mie, Japan) in Iga (34°70′N, 136°13′E) using *Bipolaris oryzae* strain Iga-2 (MAFF245177) in 2018, 2019, 2020, and 2024. To enhance disease pressure in the test field, plants of the susceptible rice cultivar ‘Mienoyume’, which had been previously infected with BS, were uniformly transplanted as an inoculum source at 30 cm inter-row and 30 cm intra-row spacing. Test materials were planted between these inoculum plants. Preparation of infected ‘Mienoyume’ seedlings for use as inoculum plants was conducted as follows. The *Bipolaris oryzae* strain was cultured on PDA plates, and a conidial suspension was prepared at a concentration of 8–10 × 10^4^ spores/mL. A surfactant (Tween 20) was added to the suspension at a final concentration of 0.02%, and 40 mL of this suspension was sprayed onto 20-day-old ‘Mienoyume’ seedlings grown in seedling trays (30 cm × 60 cm). Inoculation was performed between 18:00 and 20:00, after which the trays were kept overnight (approximately 12 hours) in a germination chamber set at 25°C under humid conditions. The trays were then removed and maintained in an unheated glasshouse at ambient temperature until transplanting. The test year(s) and number of replicates of each plant material are described above. BS disease was scored from 0 (no incidence) to 9 (severe), and the average value of two plants per replicate was used as the disease score. Score classes were defined based on the proportion of expanding lesions relative to the total number of lesions on the leaf blade, as well as the lesion area ratio (Matsumoto *et al.* 2016). A score of “3” corresponds to a small proportion of expanding lesions with a lesion area ratio of 3–5%; a score of “5” corresponds to approximately half of the lesions being expanding with a lesion area ratio of 10–15%; and a score of “7” corresponds to about three-quarters of the lesions being expanding with a lesion area ratio of 20–30%. Days to heading (DTH), which was calculated as the number of days from sowing to heading, was also examined. The dates of sowing and transplanting plant materials were as follows: 11 and 29 May 2018, 9 and 30 May 2019, 7 and 29 May 2020, and 14 and 31 May 2024. Disease scores were recorded on 21 September 2018, 16 September 2019, 11 September 2020, and 19 September 2024.

### DNA marker analysis, linkage mapping, and QTL analysis

We extracted total DNA from leaves by using the CTAB method (Murray and Thompson 1980). To survey the genotypes, we used SSR (McCouch *et al.* 2002) and single-nucleotide polymorphism (SNP) markers. SSR and SNP analysis was performed as described by Sato *et al.* (2015). Four SSR markers showing polymorphism between ‘Koshihikari’ and ‘SL2524’ and eight showing polymorphism between ‘Koshihikari’ and ‘SL3237’ were used to genotype the F_2_ plants. The genotypes of sub-CSSLs were investigated by using five polymorphic SSR markers in sub-CSSLs of ‘SL2524’ and eight in sub-CSSLs of ‘SL3237’. The genotypes of 107 RILs in the F_6_ generation derived from the cross ‘IR58’/‘Mienoyume’ were investigated by using 85 SNP markers distributed across the 12 rice chromosomes (Supplemental Table 1). NILs were developed through MAS by using the SSR markers described above.

The linkage map was constructed in MAPMAKER/EXP v. 3.0 software (Lander *et al.* 1987). Genetic distances were calculated by the Kosambi function. QTL analysis was performed in Windows QTL Cartographer v. 2.5 software (Wang *et al.* 2006) with the default composite interval mapping and control parameters, standard model 6, five control markers, a 10-cM window size, and the forward and backward regression model. The genome-wide threshold value (α = 0.05) was used to detect putative QTLs based on the results of 1000 permutations.

## Results

### Selection of BS-resistant lines from two sets of CSSLs

**Fig. 1.**
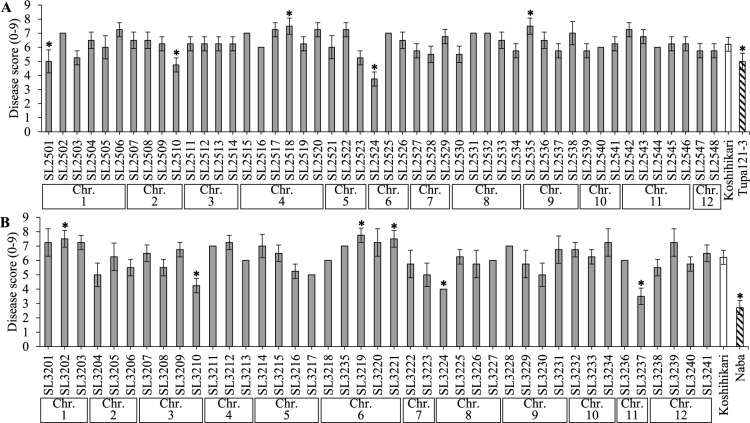
Brown sopt (BS) disease scores of two sets of CSSLs (gray bars); the recurrent parent, ‘Koshihikari’ (white bars); and the donor parents, ‘Tupa 121-3’ and ‘Naba’ (stripe bars). Error bars indicate SD. Chromosome (Chr.) numbers below the *x* axis indicate the main (A) ‘Tupa 121-3’ or (B) ‘Naba’ segment within each CSSL. *Significant difference between ‘Koshihikari’ and the CSSL or donor parent at the 5% level by Dunnett’s test.

**Fig. 2.**
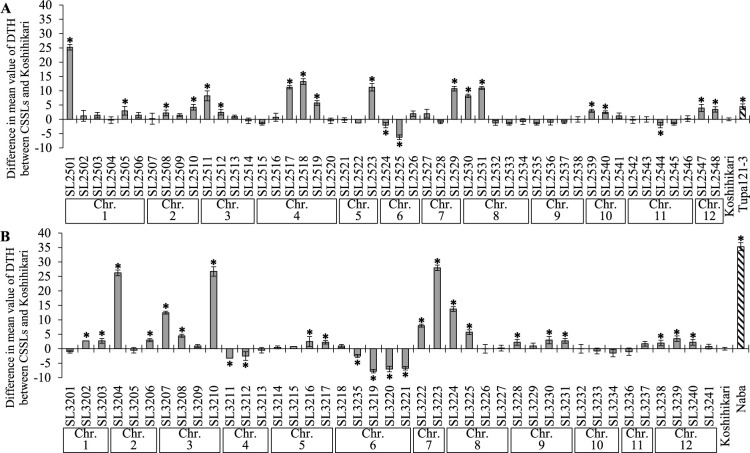
Difference in mean value of days to heading (DTH) between two sets of CSSLs or the donor parents (‘Tupa 121-3’ and ‘Naba’ [stripe bars]) and the recurrent parent, ‘Koshihikari’. Error bars indicate SD. Chromosome (Chr.) numbers below the *x* axis indicate the main (A) ‘Tupa 121-3’ and (B) ‘Naba’ segment within each CSSL. *Significant difference between ‘Koshihikari’ and the CSSL or ‘Koshihikari’ and the donor parent at the 5% level by Dunnett’s test.

In 2018, ‘Koshihikari’ had a mean BS disease score of 6.2, ‘Tupa 121-3’ of 5.0, and ‘Naba’ of 2.7 (Fig. 1). Within the two sets of CSSLs derived from these resistant cultivars, the disease scores of ‘SL2501’, ‘SL2510’, and ‘SL2524’ from the KT-CSSLs and ‘SL3210’, ‘SL3224’ and ‘SL3237’ from the KN-CSSLs were significantly lower than that of ‘Koshihikari’. Among these six CSSLs, the DTH values of ‘SL2501’, ‘SL2510’, ‘SL3210’, and ‘SL3224’ were significantly higher than that of ‘Koshihikari’ (Fig. 2). A previous study using the same evaluation method as here found significant negative correlations between DTH and BS disease score (Matsumoto *et al.* 2017b), indicating that BS resistance is often correlated with later heading. Therefore, among the CSSLs with lower BS disease scores than ‘Koshihikari’, we evaluated ‘SL2524’, which had a lower DTH value than ‘Koshihikari’, and ‘SL3237’, which had a comparable DTH value, for their potential as CSSLs with resistance to BS without an undesirable delay in heading date. We detected genetic polymorphisms between ‘Koshihikari’ and ‘SL2524’ on chr. 6 (0.00–7.65 Mbp) and between ‘Koshihikari’ and ‘SL3237’ on chr. 11 (4.00–28.98 Mbp) (Nagata *et al.* 2023).

### QTL analysis for BS resistance using F_2_ populations derived from crosses ‘Koshihikari’/‘SL2524’ and ‘Koshihikari’/‘SL3237’

**Fig. 3.**
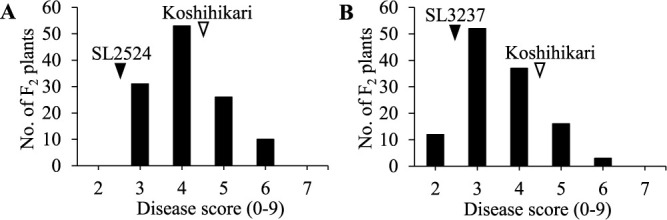
Frequency distribution of BS disease score in F_2_ plants of (A) ‘Koshihikari’/‘SL2524’ and (B) ‘Koshihikari’/‘SL3237’. Arrowheads indicate the mean values for ‘Koshihikari’, ‘SL2524’, and ‘SL3237’.

To confirm the BS resistance QTLs detected in the CSSL analysis, we developed F_2_ populations derived from crosses between ‘Koshihikari’ and BS-resistant CSSLs (‘SL2524’ and ‘SL3237’) in 2020. ‘SL2524’ had a mean BS disease score of 2.5, ‘SL3237’ of 2.5, and ‘Koshihikari’ of 4.6, and the BS disease scores in the two F_2_ populations were normally distributed (Fig. 3). There were no significant correlations between BS disease scores and DTH in either population (‘Koshihikari’/‘SL2524’, *r* = –0.018, ns; ‘Koshihikari’/‘SL3237’, *r* = –0.152, ns).

**Fig. 4.**
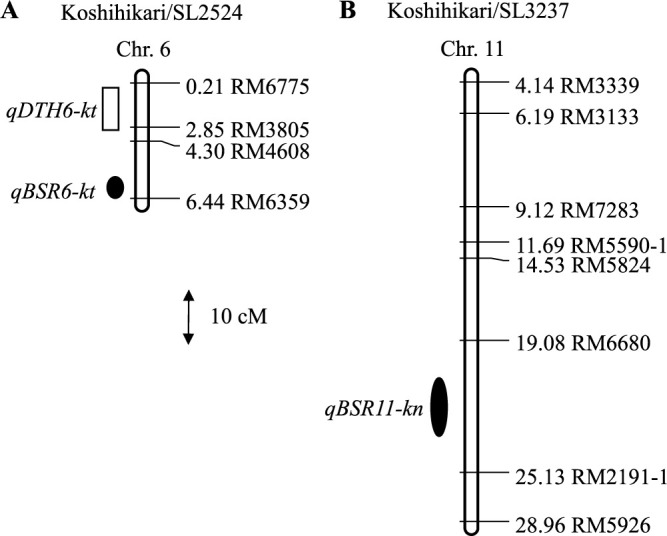
QTL regions on each chromosome (Chr.) associated with brown spot (BS) resistance and days to heading (DTH) detected in two F_2_ populations. Positions (Mbp) are indicated to the left of the SSR marker names according to the ‘Nipponbare’ pseudomolecule (IRGSP-1.0). Symbols represent QTLs with 1-LOD confidence intervals for ● BS resistance and □ DTH.

Linkage maps of chrs. 6 and 11 were constructed by genotyping the two F_2_ populations with 12 SSR markers (Fig. 4). Two QTLs for BS resistance, designated *qBSR6-kt* (from ‘SL2524’ [‘Tupa 121-3’]) and *qBSR11-kn* (from ‘SL3237’ [‘Naba’]), were mapped to genomic regions that overlapped with those identified using CSSLs (Table 1, Figs. 1, 4). The ‘SL2524’ allele explained 40.6% and the ‘SL3237’ allele 48.0% of total phenotypic variation (Table 1). At *qDTH6-kt*, a QTL from ‘SL2524’ (‘Tupa 121-3’) associated with DTH, the ‘SL2524’ allele promoted earlier heading (Table 1, Fig. 4).

### Confirming QTLs for BS resistance using sub-CSSLs

In 2020, we evaluated sub-CSSLs representing six genotypes on the ‘SL2524’ (‘Tupa 121-3’) genotype segment and sub-CSSLs representing eight genotypes on the ‘SL3237’ (‘Naba’) genotype segment (Tables 2, 3). Among the six sub-CSSLs derived from KT-CSSLs, three (‘1-6’, ‘1-16’, and ‘2979’) had low disease scores (3.3 to 3.7), whereas the other three (‘2980’, ‘2-11’, and ‘3-2’) had high disease scores (4.3 to 6.0; Table 2). These two phenotypic groups were associated with the genotype at RM6359, the SSR marker most closely linked to *qBSR6-kt*: the low-disease group was homozygous for the ‘SL2524’ (‘Tupa 121-3’) allele and the high-disease group was homozygous for the ‘Koshihikari’ allele. Among the eight sub-CSSLs derived from KN-CSSLs, three sub-CSSLs (‘4-9’, ‘4-11’, and ‘8-15’) had low disease scores (3.0 to 3.3), whereas the other five (‘7-8’, ‘5-20’, ‘6-18’, ‘7-6’, and ‘3034’) had high disease scores (4.7 to 5.3; Table 3). These two phenotypic groups were associated with genotype at RM2191-1, the SSR marker most closely linked to *qBSR11-kn*: the low-disease group was homozygous for the ‘SL3237’ (‘Naba’) allele and the high-disease group was homozygous for the ‘Koshihikari’ allele.

### QTL analysis for BS resistance using RILs derived from the cross ‘IR58’/‘Mienoyume’ and validation of QTLs from ‘IR58’

**Fig. 5.**
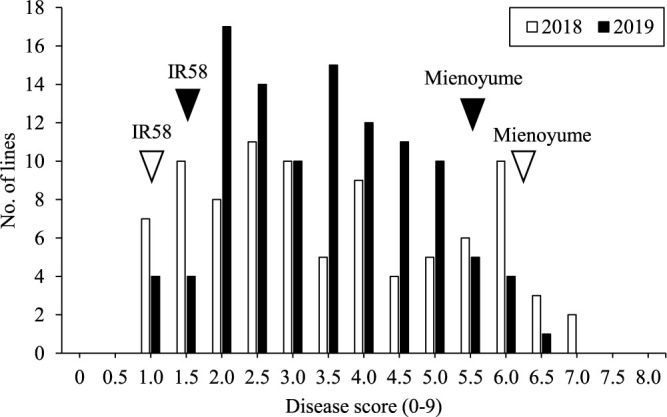
Frequency distribution of brown spot (BS) disease score in 90 RILs in 2018 and 107 in 2019. Arrowheads indicate the mean values of the parents in each year.

BS resistance differed distinctly between the parental cultivars ‘IR58’ and ‘Mienoyume’ in both 2018 and 2019, with mean BS disease scores of 1.3 and 5.9, respectively, over the two years (Fig. 5). The disease scores of the RIL population were normally distributed in both years. The RIL population exhibited transgressive segregation in both directions, which showed that BS resistance was a quantitative trait (Fig. 5). There was no significant correlation between disease score and DTH in either year (*r* = –0.15, *p* = 0.16 in 2018; *r* = –0.11, *p* = 0.26 in 2019).

**Fig. 6.**
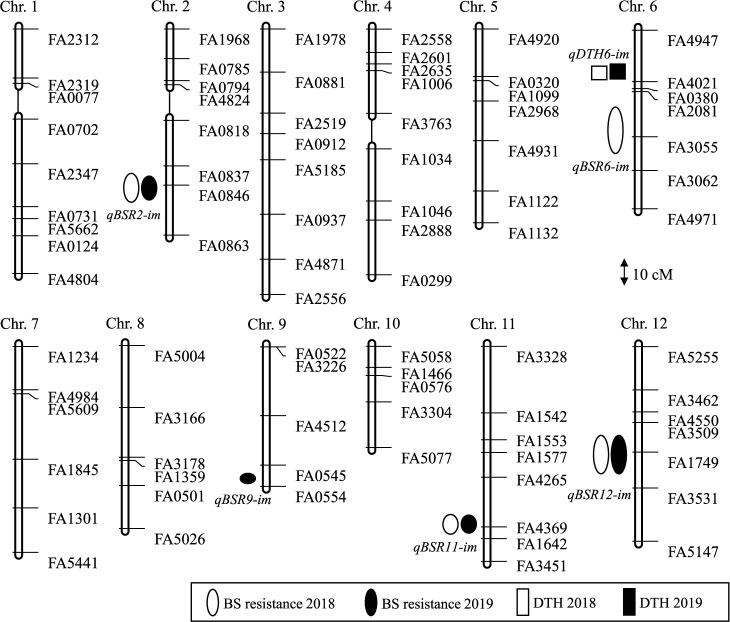
Genetic linkage map of 85 SNP markers and locations of QTLs for BS resistance and days to heading (DTH) detected in 107 RILs. Symbols represent QTL positions for ○● BS resistance and □■ DTH, and their lengths correspond to 1-LOD confidence intervals. Detailed information about SNP markers used for this mapping is shown in Supplemental Table 1.

A linkage map consisting of 12 chromosomes was constructed by genotyping the RILs with 85 SNP polymorphic markers. The genotypes across all marker loci in the RIL population were distributed as follows: 41.5% homozygous for the ‘Mienoyume’ allele, 54.4% homozygous for the ‘IR58’ allele, 2.8% heterozygous, and 1.3% with missing data. This map covered a total genetic distance of 762.9 cM and provided linkage groups for all chromosomes, except for small gaps on chrs. 1, 2, and 4, with an average distance between adjacent markers of 9.1 cM (Fig. 6).

Five QTLs for BS resistance (*qBSR2-im*, *qBSR6-im*, *qBSR9-im*, *qBSR11-im*, and *qBSR12-im*) were identified in data from 2018 and 2019 (Fig. 6, Table 4). The loci at which the ‘IR58’ allele decreased the disease score explained 7.2%–7.3% (*qBSR2-im*), 36.6%–41.3% (*qBSR11-im*), and 6.3%–12.7% (*qBSR12-im*) of total phenotypic variation, and those at which the ‘Mienoyume’ allele decreased the disease score explained 10.6% (*qBSR6-im*) and 10.5% (*qBSR9-im*) of total phenotypic variation. All of the QTLs with resistance alleles from ‘IR58’ were detected in both years, whereas those with resistance alleles from ‘Mienoyume’ were each detected in only one year. *qBSR11-im* gave the highest percentage of variance explained (41.3% in 2018) of these five QTLs and was considered a major QTL. In addition, a QTL for DTH (*qDTH6-im*) was detected in the same region in both years, but the region was different from those of the QTLs for BS resistance (Fig. 6, Table 4).

In 2024, ‘IR58’ had a disease score of 2.8 and ‘Mienoyume’ of 5.9 (Table 5). MY11, carrying the ‘IR58’ allele at *qBSR11-im*, had a significantly lower disease score (4.4) than ‘Mienoyume’. MY2, with an ‘IR58’ allele at *qBSR2-im*, had the same disease score (6.2) as ‘Mienoyume’, and MY12, with an ‘IR58’ allele at *qBSR12-im*, had a higher (6.8) disease score. The disease score of MY2+11 (4.2), carrying ‘IR58’ alleles at both *qBSR2-im* and *qBSR11-im*, was not significantly different from that of MY11. It was revealed that *qBSR11-im* was an effective QTL for BS resistance and that pyramiding *qBSR2-im* and *qBSR11-im* did not improve resistance.

## Discussion

In this study, three resistant genetic resources—‘Tupa 121-3’, ‘Naba’, and ‘IR58’—were used. When evaluated over two years using the same method as in this study, their BS disease scores were 3.0 (resistance level: “medium to strong”), 2.7 (“medium to strong”), and 0.8 (“strong”), respectively, indicating strong resistance compared to the susceptible cultivars ‘Koshihikari’ (BS disease score: 5.2; resistance level: “weak to medium”) and ‘Mienoyume’ (6.2; “weak”) (Matsumoto *et al.* 2017a). To detect BS resistance QTLs, we used two sets of CSSLs and a set of RILs. CSSLs have been used for the analysis of complex agronomic traits, resulting in the identification of numerous novel QTLs (Nagata *et al.* 2023). We used CSSLs developed by Nagata *et al.* (2023), which contain chromosome segments from BS-resistant cultivars ‘Tupa 121-3’ and ‘Naba’, and detected seven BS resistance QTLs (Tables 1, 4). Of these, five QTLs (*qBSR6-kt*, *qBSR11-kn*, *qBSR2-im*, *qBSR11-im*, and *qBSR12-im*) were derived from resistant cultivars, and two (*qBSR6-im* and *qBSR9-im*) were derived from susceptible cultivars. In a previous study, we identified multiple BS resistance QTLs derived from *indica* cultivar ‘Dawn’ (Ota *et al.* 2021). One of these, *qBSR6-kd*, was located close to *qBSR6-kt*, which was derived from ‘Tupa 121-3’ and identified here. Notably, while *qBSR6-kd* was not confirmed to confer BS resistance in our previous study (Ota *et al.* 2021), *qBSR6-kt* gave effective BS resistance, as demonstrated by the results from the sub-CSSLs (Table 2). These results indicate that *qBSR6-kt* is a novel QTL that could be used in breeding programs. Katara *et al.* (2010) identified 11 QTLs in 154 doubled-haploid lines derived from a cross between ‘CT9993’ (resistant) and ‘IR62266’ (moderately susceptible) in India: three of these QTLs, on chrs. 2 (*BSq2.2v* from IR62266), 11 (*BSq11.2v* from IR62266), and 12 (*BSq12.1v* from CT9993), were located at loci similar to the QTLs at which the ‘IR58’ allele decreased BS disease score here. The marker intervals of *qBSR11-kn* (Table 1) and *qBSR11-im* (Table 4) were similar to that of BS resistance QTL *bsr1* derived from ‘Tadukan’ (Matsumoto *et al.* 2021, Sato *et al.* 2015). Mizobuchi *et al.* (2024) reported that *bsr1* is located within a 23.7–24.2 Mbp interval on chr. 11. In our previous study, *qBSR11-kc*, derived from BS-resistant cultivar ‘CH45’, was also detected in the same region, spanning 21.5–25.1 Mbp on chr. 11 (Matsumoto *et al.* 2017b). It is noteworthy that BS resistance QTLs from multiple cultivars originating in different parts of the world have been detected in the same region on chr. 11. For example, ‘Naba’ (donor of *qBSR11-kn*), is native to India, while ‘IR58’ (donor of *qBSR11-im*) and ‘Tadukan’ (donor of *bsr1*) are native to the Philippines. In addition, BS resistance of ‘CH45’ (donor of *qBSR11-kc*) was confirmed in India (Misra 1985). Moreover, *bsr1* gave resistance to multiple isolates of BS fungus collected from different regions in Japan (Matsumoto *et al.* 2021). If these QTLs represent the same BS resistance gene, the gene should confer BS resistance over a wide geographic area. Our research group is currently attempting to clone the resistance gene derived from ‘Tadukan’ in this QTL region on chr. 11. Moreover, we will examine the allelic variation surrounding the isolated resistance gene in the previously described donor cultivars, which carry BS resistance QTLs mapped to the *bsr1* locus, to determine whether these QTLs are due to the same causal gene as *bsr1*.

Significant negative correlations between DTH and BS disease score have been reported (Matsumoto *et al.* 2017a, 2017b). Here, we detected two QTLs for DTH, *qDTH6-kt* and *qDTH6-im*; at these QTLs, the ‘SL2524’ and the ‘IR58’ allele, respectively, decreased DTH (Table 1, 4). This result shows that BS resistance confirmed here was not a pleiotropic effect of delayed heading.

Three of the QTLs detected here—*qBSR6-kt* (derived from ‘Tupa 121-3’), *qBSR11-kn* (derived from ‘Naba’), and *qBSR11-im* (derived from ‘IR58’)—were each confirmed to have BS resistance effects in the genetic background of a BS-susceptible *japonica* cultivar (‘Koshihikari’ or ‘Mienoyume’) (Tables 2, 3, 5). In our previous studies, only *bsr1* has been confirmed to confer resistance in a *japonica* genetic background. *qBSR11-kn* and *qBSR11-im* on chr. 11 are in the same region as *bsr1* and probably represent the same gene. In contrast, *qBSR6-kt* on chr. 6 is a novel QTL that could be used in breeding programs in combination with *bsr1*. Furthermore, similar to *bsr1*, future studies on *qBSR6-kt* will focus on narrowing down the causal gene(s) through transcriptomic analysis (e.g., RNA-seq) under *Bipolaris oryzae* infection conditions, combined with polymorphism screening (e.g., SNPs and indels) among parental lines and NILs with contrasting resistance. These efforts will support the fine-mapping of the QTL and the identification of strong candidate genes.

## Author Contribution Statement

YO performed experiments for QTL analysis and developed plant materials for verification of QTLs. KM developed plant materials for verification of QTLs. YO and KM wrote the manuscript with assistance from the other authors. YH performed experiments for verification of QTLs. SO and DN performed field evaluations. RM and HS performed SNP analysis, supervised the research, and revised the manuscript.

## Supplementary Material

Supplemental Tables

## Figures and Tables

**Table 1.** Putative QTLs for brown spot (BS) resistance and days to heading (DTH) in two F_2_ populations

Population	Trait	QTL	Chr*^a^*	Marker interval*^b^*	Physical interval (Mbp)	LOD*^c^*	Variance explained (% of total)	Additive effect*^d^*	LOD threshold
Koshihikari/SL2524	BS resistance*^e^*	*qBSR6-kt*	6	RM4608–RM6359	4.3–6.4	13.1	40.6	–0.6	1.8
	Days to heading	*qDTH6-kt*	6	RM6775–RM3805	0.2–2.9	4.2	14.8	–0.6	1.8
Koshihikari/SL3237	BS resistance	*qBSR11-kn*	11	RM6680–RM2191-1	19.1–25.1	12.5	48.0	–0.9	2.2

*^a^* Chromosome. *^b^* The nearest markers are underlined. *^c^* Logarithm of odds score. *^d^* Negative values mean that the ‘SL2524’ (‘Tupa 121-3’) or ‘SL3237’ (‘Naba’) allele decreased the trait value. *^e^* BS resistance was assessed by disease score: lower scores indicate greater resistance.

**Table 2.** Genotypes of five SSR markers at *qBSR6-kt* locus and brown spot (BS) disease scores in the sub-CSSLs derived from KT-CSSLs

Line	Genotype of SSR marker*^a^*					Disease score*^c^* (0–9)
	RM6775 (0.21)*^b^*	RM3805 (2.85)	RM4608 (4.30)	RM6359 (6.44)	RM3431-1 (8.75)	
1-6	B	B	B	B	A	3.3 ± 0.6**
1-16	A	B	B	B	A	3.7 ± 0.6**
2979	A	A	A	B	A	3.7 ± 0.6**
2980	B	A	A	A	A	4.3 ± 0.6
2-11	B	B	A	A	A	6.0 ± 0.0
3-2	B	B	B	A	A	5.3 ± 0.6
Koshihikari	A	A	A	A	A	5.3 ± 0.6
SL2524	B	B	B	B	A	3.3 ± 0.6***

*^a^* Genotypes of the SSR markers are repesented by A (white) for homozygous ‘Koshihikari’ allele and B (gray shading) for homozygous ‘SL2524’ (‘Tupa 121-3’) allele. *^b^* Numbers in parentheses below the SSR marker names indicate their physical positions (Mbp) on chromosome 6, according to the ‘Nipponbare’ pseudomolecule (IRGSP-1.0). *^c^* Disease scores for BS are shown as mean ± standard deviation (SD). Asterisks indicate significant difference between ‘Koshihikari’ and the sub-CSSL at the **1% and ***0.1% level by Dunnett’s test.

**Table 3.** Genotypes of eight SSR markers at *qBSR11-kn* locus and brown spot (BS) disease scores in the sub-CSSLs derived from KN-CSSLs

Line	Genotype of SSR marker*^a^*								Disease score*^c^* (0–9)
	RM3339 (4.14)*^b^*	RM3133 (6.19)	RM7283 (9.12)	RM5590-1 (11.69)	RM5824 (14.53)	RM6680 (19.08)	RM2191-1 (25.13)	RM5926 (28.96)	
4-9	A	A	B	B	B	B	B	B	3.3 ± 0.6***
4-11	A	A	A	A	A	B	B	B	3.0 ± 0.0***
8-15	A	A	A	A	A	A	B	B	3.0 ± 0.0***
7-8	B	B	B	B	B	B	A	A	4.7 ± 0.6
5-20	B	B	B	B	B	A	A	A	4.7 ± 0.6
6-18	B	B	A	A	A	A	A	A	5.3 ± 0.6
7-6	B	B	A	A	A	A	A	A	4.7 ± 0.6
3034	A	B	A	A	A	A	A	A	4.7 ± 0.6
Koshihikari	A	A	A	A	A	A	A	A	5.3 ± 0.6
SL3237	B	B	B	B	B	B	B	B	3.3 ± 0.6***

*^a^* Genotypes of the SSR markers are represented by A (white) for homozygous ‘Koshihikari’ allele and B (gray shading) for homozygous ‘SL3237’ (‘Naba’) allele. *^b^* Numbers in parentheses below the SSR markers indicate their physical position (Mbp) on chromosome 11, according to the ‘Nipponbare’ pseudomolecule (IRGSP-1.0). *^c^* Disease scores for BS are shown as mean ± SD. Asterisks indicate significant difference between ‘Koshihikari’ and the sub-CSSL at the ***0.1% level by Dunnett’s test.

**Table 4.** Putative QTLs for brown spot (BS) resistance and days to heading (DTH)

Trait	Year	QTL	Chr*^a^*	Marker interval*^b^*	Physical interval (Mbp)	LOD*^c^*	Variance explained (% of total)	Additive effect*^d^*	LOD threshold
BS resistance*^e^*	2018	*qBSR2-im*	2	FA0837–FA0846	28.2–31.5	3.3	7.3	–0.5	2.8
		*qBSR6-im*	6	FA2081–FA3055	19.6–24.2	3.5	10.6	0.6	2.8
		*qBSR11-im*	11	FA4265–FA4369	18.9–23.4	13.0	41.3	–1.2	2.8
		*qBSR12-im*	12	FA1749–FA3531	20.8–24.2	3.9	12.7	–0.7	2.8
	2019	*qBSR2-im*	2	FA0846–FA0863	31.5–35.9	4.0	7.2	–0.4	2.7
		*qBSR9-im*	9	FA0545–FA0554	14.8–18.5	5.5	10.5	0.4	2.7
		*qBSR11-im*	11	FA4265–FA4369	18.9–23.4	14.9	36.6	–0.8	2.7
		*qBSR12-im*	12	FA1749–FA3531	20.8–24.2	2.8	6.3	–0.3	2.7
Days to heading	2018	*qDTH6-im*	6	FA4947–FA4021	6.1–10.7	23.9	60.1	–16.6	3.5
	2019	*qDTH6-im*	6	FA4947–FA4021	6.1–10.7	24.6	52.9	–12.7	5.3

*^a^* Chromosome. *^b^* The nearest markers are underlined. *^c^* Logarithm of odds score. *^d^* Negative values mean that the ‘IR58’ allele decreased the trait value. *^e^* BS resistance was assessed by disease score: lower scores indicate higher resistance.

**Table 5.** Genotypes and brown spot (BS) disease scores in 2024 in the NILs carrying the ‘IR58’ allele at each detected QTL locus

NIL group	Generation	Genotype of SSR marker*^a^*											No. of lines	Disease score*^c^* (0–9)
		*qBSR2-im*			*qBSR6-im*	*qBSR9-im*		*qBSR11-im*			*qBSR12-im*			
		RM13958 (31.2)*^b^*	RM3850 (35.4)		RM20366 (24.2)	RM6235 (16.7)		RM5349 (19.7)	RM27073 (24.0)		RM28305 (20.0)	RM3331 (23.5)		
MY2	BC_3_F_4_	B	B		A	A		A	A		A	A	3	6.2 ± 0.4 bc
MY11	BC_3_F_4_	A	A		A	A		B	B		A	A	3	4.4 ± 0.1 a
MY12	BC_3_F_4_	A	A		A	A		A	A		B	B	3	6.8 ± 0.3 c
MY2+11	F_3_	B	B		A	A		B	B		A	A	3	4.2 ± 0.2 a
Mienoyume	–	A	A		A	A		A	A		A	A	3	5.9 ± 0.1 b
IR58	–	B	B		B	B		B	B		B	B	1	2.8–

*^a^* Genotypes are represented by A (white) for homozygous ‘Mienoyume’ allele and B (gray shading) for homozygous ‘IR58’ allele. *^b^* Numbers in parentheses below the SSR markers indicate their physical map position (Mbp) on the chromosomes, according to the ‘Nipponbare’ pseudomolecule (IRGSP-1.0). *^c^* Disease scores for BS are shown as mean ± SD of three lines, except for ‘IR58’. Disease scores followed by the same letter are not significantly different at the 5% level by Tukey–Kramer test.
